# Interleukin-6, Tocilizumab and Atherosclerosis. Comment on Gerasimova et al. Interleukin-6: Cardiovascular Aspects of Long-Term Cytokine Suppression in Patients with Rheumatoid Arthritis. *Int. J. Mol. Sci.* 2024, *25*, 12425

**DOI:** 10.3390/ijms26072952

**Published:** 2025-03-25

**Authors:** Christian Saleh, Hrvoje Budincevic

**Affiliations:** 1 Independent Researcher, 4056 Basel, Switzerland; 2 Department of Neurology, Sveti Duh University Hospital, 10000 Zagreb, Croatia; hbudincevic@gmail.com; 3 Department of Neurology and Neurosurgery, Faculty of Medicine Osijek, J. J. Strossmayer University of Osijek, 31000 Osijek, Croatia

Gerasimova et al. published in a recent Special Issue a study titled “Interleukin-6: Cardiovascular Aspects of Long-Term Cytokine Suppression in Patients with Rheumatoid Arthritis” [1].

This prospective study, with a 275-week follow-up period, investigated in 37 patients (86% female, median age of 57 years) with rheumatoid arthritis (RA) the effect of IL-6 receptor blocker tocilizumab (TCZ) therapy on cardiovascular risk (CVR) factors and atherosclerosis [1]. As a surrogate marker for pre-clinical atherosclerosis, carotid intima-media thickness (cIMT) was used [1]. The study found that during the 5-year follow-up period, there was no increase in cIMT (pre-treatment cIMT bilateral mean 0.85 mm, post-treatment after 265 weeks cIMT bilateral mean 1.05 mm) [1]. Some comments (points 1–7) are needed to evaluate the results of this study in a more exhaustive way. 1. Definition of cIMT: While the authors define plaque, they do not provide any definition for cIMT nor any cut-off to define pathologically increased cIMT [1]. It remains, therefore, unclear if the authors also included in their cIMT measurement plaque measurements and, therefore, performed composite measures. Composite measures, i.e., including cIMT and plaque, are not recommended, as cIMT and plaque are distinct entities [2,3]. Johnsen and Mathiesen wrote, “The pathologic processes leading to intima-media thickening and to plaque formation … may not be similar and plaque and intima-media thickening may reflect different biological aspects of atherogenesis with distinctive relations to clinical vascular disease” [3]. 2. Location of cIMT measurement: The authors did not specify where within the carotid artery (CA) tree the cIMT measurement occurred [1]. As there is no consensus on the measurement protocol, the applied protocol needs detailing: There are two major guidelines for cIMT measurement, namely a) a one-site location (generally performed at the distal wall of the common CA (CCA)) and b) a multi-site location measurement, including different segments of the CA tree, e.g., proximal/distal walls of CCA, bifurcation, and internal CA [2,4]. A multi-site measurement will capture the asymmetric nature of atherosclerosis in a more accurate way, providing, therefore, a more precise cIMT estimate [5]. 3. Follow-up visit: As the authors did not provide any information as to where within the CA tree the cIMT was measured, it is also not possible to evaluate where within the CA tree the follow-up cIMT measures were performed [1]. To determine the rate of cIMT change over time, a delicate and difficult task, it is of critical importance to document precisely the baseline CA section used for the cIMT measurement [2]. If this did not occur, the follow-up measurement was likely made in a different area, also if adjacent to the original segment, but with potentially different wall characteristics. The determined rate of change will be unavoidably inaccurate. 4. Inter-rater variability: As the follow-up extended over 5 years, it is of critical importance to know if the same operator or different operators performed the measurements [1]. The intra- and inter-operator variability needs to be known [6]. This critical information/data were not provided [1]. 5. Measurement method: The measurement method (manual or software-assisted) needs to be reported [7]. Secil et al. [8] wrote in their paper “Automated measurement of intima-media thickness of carotid arteries in ultrasonography by computer software” the following: “Manual measurement methods are generally used to measure the IMT values sonographically, but results show variations secondary to subjective parameters when manual measurement methods are employed. Additionally, studies have demonstrated that CCA IMT values may show interobserver variability in repeated measurements. To decrease the interobserver variability during the IMT measurements, computer software programs were developed that allow automated measurements… The interobserver correlation of automated measurements is higher than manual measurements. Dedicated software may be proposed to reduce the measurement errors” [8]. 6. Cardiac synchronization: The measurement of cIMT needs to be synchronized with the cardiac cycle, namely the end-diastolic phase [2,9,10]. Equally, this critical information was not provided [1]. Mean cIMT differences between the systolic and diastolic phases of 0.041 mm were reported (given lumen-diameter changes during the cardiac cycle) [9]. Not synchronizing cIMT with the end-diastolic phase will lead to random measurements in both cardiac phases, rendering the measurements cross-sectionally as well as longitudinally incomparable. 7. Comparison with other studies: The authors compared their cIMT results with several other studies, i.e., Chen et al. [11], Kume et al. [12], and Okazaki et al. [13]. However, these comparisons cannot be made a) as the measurement protocol by Gerasimova et al. [1] was not detailed and b) because the applied methodologies of the cited studies by Chen et al. [11], Kume et al. [12], and Okazaki et al. [13] differ. Studies that are methodologically diverse are not comparable. Conclusion: When applying cIMT as a surrogate marker, high-precision measurements are required, and a detailed measurement protocol needs to be in place. Normal cIMT is expressed at a sub-millimeter level (e.g., 0.6–0.8 mm); the slightest measurement inaccuracies or a sub-optimal defined measurement protocol that does not consider the complexity of atherosclerotic presentation easily result in inaccurate measures misclassifying subjects into different cIMT categories, from which important clinical and pathophysiological conclusions are drawn. The cIMT results and related conclusions of Gerasimova et al.’s study [1] should be analyzed within the context of the abovementioned methodological limitations.

## References

[1] Gerasimova E.V., Popkova T.V., Kirillova I.G., Gerasimova D.A., Nasonov E.L., Lila A.M. (2024). Interleukin-6: Cardiovascular Aspects of Long-Term Cytokine Suppression in Patients with Rheumatoid Arthritis. Int. J. Mol. Sci..

[2] Touboul P.J., Hennerici M.G., Meairs S., Adams H., Amarenco P., Bornstein N., Csiba L., Desvarieux M., Ebrahim S., Woo K.S. (2012). Mannheim carotid intima-media thickness and plaque consensus (2004–2006–2011). An update on behalf of the advisory board of the 3rd, 4th and 5th Watching the Risk Symposia, at the 13th, 15th and 20th European Stroke Conferences, Mannheim, Germany, 2004, Brussels, Belgium, 2006, and Hamburg, Germany, 2011. Cerebrovasc. Dis..

[3] Johnsen S.H., Mathiesen E.B. (2009). Carotid plaque compared with intima-media thickness as a predictor of coronary and cerebrovascular disease. Curr. Cardiol. Rep..

[4] Bots M.L., Evans G.W., Riley W.A., Grobbee D.E. (2003). Carotid intima-media thickness measurements in intervention studies: Design options, progression rates, and sample size considerations: A point of view. Stroke.

[5] Tajik P., Meijer R., Duivenvoorden R., Peters S.A.E., Kastelein J.J., Visseren F.J., Crouse J.R., Palmer M.K., Raichlen J.S., Grobbee D.E. (2012). Asymmetrical distribution of atherosclerosis in the carotid artery: Identical patterns across age, race, and gender. Eur. J. Prev. Cardiol..

[6] Delaney J.A.C., Scherzer R., Polak J., Biggs M.L., Kronmal R., Chen H., Sidney S., Grunfeld C. (2010). Effect of inter-reader variability on outcomes in studies using carotid intima media thickness quantified by carotid ultrasonography. Eur. J. Epidemiol..

[7] Polak J.F., Funk L.C., O’Leary D.H. (2011). Inter-Reader Differences in Common Carotid Artery Intima-Media Thickness. J. Ultrasound Med..

[8] Seçil M., Altay C., Gülcü A., Ceçe H., Goktay A., Dicle O. (2005). Automated measurement of intima-media thickness of carotid arteries in ultrasonography by computer software. Diagn. Interv. Radiol..

[9] Polak J.F., Johnson C., Harrington A., Wong Q., O’Leary D.H., Burke G., Yanez N.D. (2012). Changes in carotid intima-media thickness during the cardiac cycle: The multi-ethnic study of atherosclerosis. J. Am. Heart Assoc..

[10] Polak J.F., Meisner A., Pencina M.J., Wolf P.A., D’Agostino R.B. (2012). Variations in Common Carotid Artery Intima-Media Thickness (cIMT) during the Cardiac Cycle: Implications for Cardiovascular Risk Assessment. J. Am. Soc. Echocardiogr..

[11] Chen D.-Y., Chen Y.-M., Hsieh T.-Y., Hsieh C.-W., Lin C.-C., Lan J.-L. (2015). Significant effects of biologic therapy on lipid profiles and insulin resistance in patients with rheumatoid arthritis. Arthritis Res. Ther..

[12] Kume K., Amano K., Yamada S., Hatta K., Ohta H., Kuwaba N. (2011). Tocilizumab monotherapy reduces arterial stiffness as effectively as etanercept or adalimumab monotherapy in rheumatoid arthritis: An open-label randomized controlled trial. J. Rheumatol..

[13] Okazaki S., Sakaguchi M., Miwa K., Furukado S., Yamagami H., Yagita Y., Mochizuki H., Kitagawa K. (2014). Association of Interleukin-6 With the Progression of Carotid Atherosclerosis. Stroke.

